# Acute effect of cycling intervention on carotid arterial hemodynamics: basketball athletes versus sedentary controls

**DOI:** 10.1186/1475-925X-14-S1-S17

**Published:** 2015-01-09

**Authors:** Hai-Bin Liu, Wen-Xue Yuan, Kai-Rong Qin, Jie Hou

**Affiliations:** 1 Department of Physical Education, Dalian University of Technology, Dalian, 116024, China; 2 Department of Biomedical Engineering, Faculty of Electronic Information and Electrical Engineering, Dalian University of Technology, Dalian, 116024,China

**Keywords:** Basketball athletes, cycling intervention, arterial stiffness, hemodynamics, common carotid artery

## Abstract

**Objective:**

To compare the acute effects of a cycling intervention on carotid arterial hemodynamics between basketball athletes and sedentary controls.

**Methods:**

Ten young long-term trained male basketball athletes (BA) and nine age-matched male sedentary controls (SC) successively underwent four bouts of exercise on a bicycle ergometer at the same workload. Hemodynamic variables at right common carotid artery were determined at rest and immediately following each bout of exercise. An ANCOVA was used to compare differences between the BA and SC groups at rest and immediately following the cycling intervention. The repeated ANOVA was used to assess differences between baseline and each bout of exercise within the BA or SC group.

**Results:**

In both groups, carotid hemodynamic variables showed significant differences at rest and immediately after the cycling intervention. At rest, carotid arterial stiffness was significantly decreased and carotid arterial diameter was significantly increased in the BA group as compared to the SC group. Immediately following the cycling intervention, carotid arterial stiffness showed no obvious changes in the BA group but significantly increased in the SC group. It is worth noting that while arterial stiffness was lower in the BA group than in the SC group, the oscillatory shear index (OSI) was significantly higher in the BA group than in the SC group both at rest and immediately following the cycling intervention.

**Conclusion:**

Long-term basketball exercise had a significant impact on common carotid arterial hemodynamic variables not only at rest but also after a cycling intervention. The role of OSI in the remodeling of arterial structure and function in the BA group at rest and after cycling requires clarification.

## Introduction

Arterial hemodynamic variables including arterial stiffness and shear stress are associated with the occurrence and progression of cardio- and cerebrovascular diseases. It is well established that arterial stiffness is a risk marker of future cardio- and cerebrovascular events such as atherosclerosis, stroke, and conventional cardiovascular diseases [1,2]. Blood flow-induced shear stress is an important mediator of arterial function [3,4].

Depending on its intensity, exercise training has been found to either reduce or increase arterial stiffness [5]. Exercise can directly induce systemic and local hemodynamic responses [6]. Vascular endothelial cells and smooth muscle cells in the blood vessels may sense these hemodynamic responses, leading to cellular responses that involve changes in cell morphology, cell function, and gene expression that are closely related to changes in arterial stiffness and diameter [7].

Regular aerobic exercise and endurance training has been documented to decrease arterial stiffness and benefit individual health [8]. Endurance training [9-11] involving activities such as cycling, rowing, distance running, and swimming decreased arterial stiffness. By contrast, the effect of resistance training on arterial stiffening has been controversial [12]. Nonetheless, resistance training has been widely recommended for the prevention of sarcopenia and osteoporosis [13]. Many studies [14-16] have shown that arterial stiffness was significantly increased after high-intensity resistance training including weight lifting, shot put, hammer, javelin, and body-building. A number of other studies [5,17], however, have indicated that arterial stiffness was significantly decreased after resistance training.

Competitive basketball is an exciting form of exercise enjoyed by more than 300 million people worldwide. Basketball training provides a unique stimulus different from the aforementioned forms of exercise. However, very little information is available concerning the effects of a combined exercise modality such as basketball training, which involves significant elements of both endurance and resistance training, on carotid arterial stiffness and associated hemodynamics.

Acute exercise intervention is an effective method of investigating the cardiovascular functional responses to external stimuli. Hoonjan et al. [14] compared the acute effects of moderate intensity aerobic exercise on arterial stiffness among endurance-trained athletes, resistance-trained athletes, and sedentary controls. To date, however, little attention has been paid to the impact of bouts of exercise on arterial stiffness in individuals who are exposed to both resistance training and endurance training in activities such as basketball exercise.

The purpose of this study was to compare the acute effects of a cycling intervention on carotid arterial hemodynamics between basketball athletes and sedentary controls. Towards this end, 10 young male basketball athletes (BA) and 9 age-matched male sedentary controls (SC) successively underwent 4 bouts of exercise on a bicycle ergometer at the same workload. Right common carotid artery stiffness and local hemodynamic variables were determined at rest and immediately following each bout of exercise. An ANCOVA with baseline score as a covariate was used to compare differences between athletes and controls. A repeated ANOVA was used to assess differences between baseline and each bout of exercise within each group. The study will provide not only hemodynamic information useful for identifying an effective form of exercise for improving arterial function, but also serve as a basis for further understanding the hemodynamic mechanisms underlying the control of arterial stiffness by exercise training.

## Methods

### Subjects

Ten young male college basketball athletes (age, 23.9 ± 1.2 years; stature, 1.90 ± 0.07 m; mass, 90.40 ± 11.57 kg; body mass index (BMI), 22.75 ± 2.57 kgm^-2^) and 9 young male healthy sedentary adults (age, 24.22 ± 2.98 years; stature, 1.74 ± 0.05 m; mass, 71.61 ± 7.69 kg; BMI, 21.62 ± 2.48 kgm^-2^) participated in this study. The basketball players had been exercising for 9 ± 2 years. The training intensity was 90 minutes/day and the duration was 4-5 days/week. The training involved endurance, speed, power, and strength elements as well as basketball skills. The control group included subjects who were not involved in any regular planned exercise program [18]. All subjects were free from hypertensive, cardiovascular, and metabolic diseases as assessed by medical history. None of the subjects were taking cardiovascular or blood pressure medications. The subjects were fully informed of the purposes, risks, and discomforts associated with the experiment before signing written informed consent. All procedures were approved by the Ethics Committee, Dalian University of Technology, China.

### Protocol of cycling intervention

**Figure 1:**
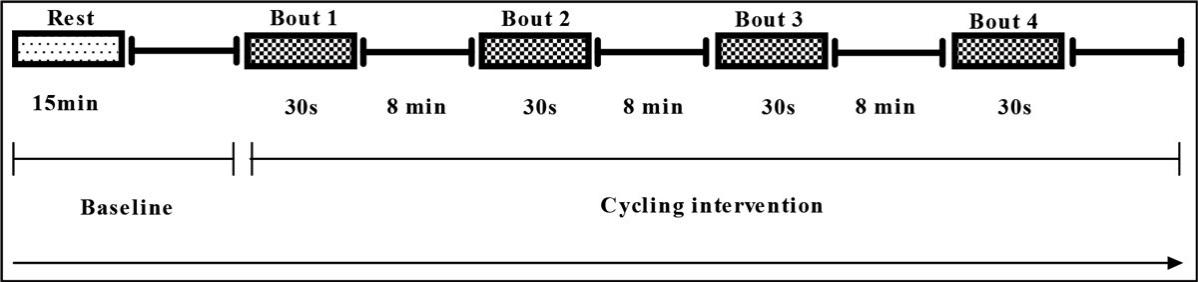
**Schematic presentation of cycling intervention protocol**.

The cycling intervention protocol is summarized in Figure 1. First, hemodynamic variables were measured after subjects lay in the supine position for ≥15 minutes. Next, subjects performed the leg cycling exercise on a bicycle ergometer (Powermax-VIII, Combi Wellness) for 4 bouts of 30 s each. The workload was set at 250 W and cadence was maintained at 60-70 RPM. Subjects resumed the supine position for measurement of hemodynamic variables after each bout of cycling. The interval between bouts was 8 min including measurement in the supine position and rest on the bicycle ergometer.

### Vascular ultrasound procedures

The arterial inner diameters and center-line blood flow velocity waveforms were acquired at the right common carotid arteries 1-2 cm proximal to the bifurcation using a color Doppler ultrasound (ProSound Alpha 7, Aloka) with no variation during each measurement. At rest and immediately following each bout of cycling, the heart rate, brachial systolic pressure (*p_s_mea_*), and diastolic pressure (*p_d_mea_*) were simultaneously measured with a cuff-type manometer (Patient Monitor PM8000, Mindray) placed on the left upper arm. All heart rate and blood pressure measurements were made in triplicate and the average of the 3 values was recorded for subsequent analysis.

### Computation of local hemodynamic parameters

#### Blood pressure

There was a linear relationship between the changes in vessel diameter and the changes in blood pressure [19] within each cardiac cycle. Therefore, blood pressure and the diameter waveforms in the carotid artery are similar. In this study, we considered the mean value of the carotid arterial pressure *p_m _*and diastolic pressure *p_d _*to be approximately equal to the mean value of the brachial pressure *p_m_mea _*and diastolic pressure *p_d_mea_*, as done in the literature [20]. The mean arterial pressure (*p_m_*) was calculated using the approximate equation:

(1) $$p_{m}=p_{m\text{\_}mea}=p_{d\text{\_}mea}+\frac{1}{3}\left(p_{s\text{\_}mea}-p_{d\text{\_}mea}\right).$$

Therefore, the carotid artery blood pressure waveform was calibrated using the brachial mean arterial *p_m _*and diastolic pressure *p_d _*(*p_d_mea_*). The maximal value of the carotid artery waveform was calculated and assumed to be the systolic pressure *p_s_*.

#### Flow rate (FR)

The FR was calculated as

(2) $$Q=2\pi {R_{0}}^{2}\int_{0}^{1}y\cdot u\left(y\right)dy,$$

where *R*_0 _is the time-averaged value of the carotid artery radius in one cardiac cycle, *y *= *r*/*R*_0 _in which *r *is the radial coordinate, and *u*(*y*) satisfies [21]

(3) $$u\left(y,t\right)=\overset{+\infty }{\underset{n=-\infty }{\sum }}\frac{J_{0}\left(\alpha_{n}j^{\frac{3}{2}}\right)-J_{0}\left(\alpha_{n}j^{\frac{3}{2}}y\right)}{J_{0}\left(\alpha_{n}j^{\frac{3}{2}}\right)-1}u\left(0,\omega_{n}\right)e^{j\omega_{n}t},$$

where *n *is the harmonic number, *J_0 _*is the 0^th^-order Bessel function of the first kind, $j=\sqrt{-1}$, $\alpha_{n}=R_{0}\sqrt{\rho \omega_{n}/)\eta }$ is the Womersley number, *ρ *is the density of blood, *η *is blood viscosity, *ω_n _*= 2*nπ f *is angular frequency, and *f *is the base frequency, while *u*(0, *ω_n_*) is the *n *harmonic component of the measured center-line velocities and satisfies

(4) $$u\left(0,t\right)=\overset{+\infty }{\underset{n=-\infty }{\sum }}u\left(0,\omega_{n}\right)e^{j\omega_{n}t}.$$

*V_max_, V_min_*, and *V_mean _*are the maximal, minimal, and mean center-line velocities after one cardiac cycle. *Q_max_*, *Q_min_*, and *Q_mean _*are the maximal, minimal, and mean blood flow FR after one cardiac cycle.

### Circumferential strain (CS)

CS was calculated using the following equation [22]:

(5) $$\text{CS = }\frac{R_{s}\text{ - }R_{d}}{R_{d}},$$

where *R_s _*and *R_d _*are maximal (systolic) and minimal (diastolic) arterial diameters.

### Wall shear stress (WSS)

WSS (*τ_w_*) was the frictional force of blood flow acting on the vascular endothelium [2] and was calculated as [21]:

(6) $$\tau_{w}=\frac{\eta }{R_{0}}\overset{+\infty }{\underset{n=-\infty }{\sum }}\frac{\alpha_{n}j^{\frac{3}{2}}J_{1}\left(\alpha_{n}j^{\frac{3}{2}}\right)}{J_{0}\left(\alpha_{n}j^{\frac{3}{2}}\right)-1}u\left(0,\omega_{n}\right)e^{j\omega_{n}t},$$

where *J_1 _*is the first order Bessel function of the first kind. *τ_w_max_*, *τ_w_min_*, and *τ_w_mean _*refer to the maximal, minimal, and mean shear stress waveforms after a cardiac cycle.

### Oscillatory shear index (OSI)

The OSI is an index that describes the shear stress acting in directions other than the direction of the temporal mean shear stress vector and was defined by Ku et al. [23] as

(7) $$\text{OSI = }\frac{1}{2}\left(\text{1 - }\frac{\left|\int_{\text{0}}^{T}\tau_{\text{w}}dt\right|}{\int_{0}^{T}\left|\tau_{\text{w}}\right|dt}\right),$$

where *T *is the period of one cardiac cycle.

### Pressure-strain elastic modulus (E_p_)

The E_P _or Peterson modulus [10] was a measure of blood vessel elasticity calculated by the following equation:

(8) $${\text{E}}_{\text{p}}\text{ = }\frac{p_{s}-p_{d}}{R_{s}-R_{d}}R_{d}.$$

### β-Stiffness index (*β*)

*β *was calculated as a means of adjusting arterial compliance for changes in distending pressure as follows [5]:

(9) $$\beta \text{ = }\frac{\text{ln}\left(p_{s}/)p_{d}\right)}{R_{s}-R_{d}}\cdot R_{d}.$$

### Data processing and statistical analysis

Local hemodynamic parameters were calculated using the above equations (1) - (9), in which blood viscosities *η *or blood densities *ρ *were set at the same values for all subjects, i.e., *η = *0.004 Pa.s and *ρ *= 1050 kg/m^3^, and the maximal harmonic number *n *was set at 20 in Eqs (3), (4), and (6).

All dynamic parameters as well as the arterial diameter were standardized by BMI [24] (kg/m^2^) and reported as (data/(kgm^-2^)). An ANCOVA with baseline score as a covariate was used for statistical analysis to compare differences between BA and SC after the cycling intervention. The repeated ANOVA was used to assess differences between baseline and each exercise bout within each group. For all analyses, significance was set at *P = *0.05. All values are presented as mean ± SD. SPSS 17.0 software (SPSS Inc., Chicago, IL, USA) was used for all analyses.

## Results

### Effect of cycling intervention on arterial stiffness and diameter

**Figure 2:**
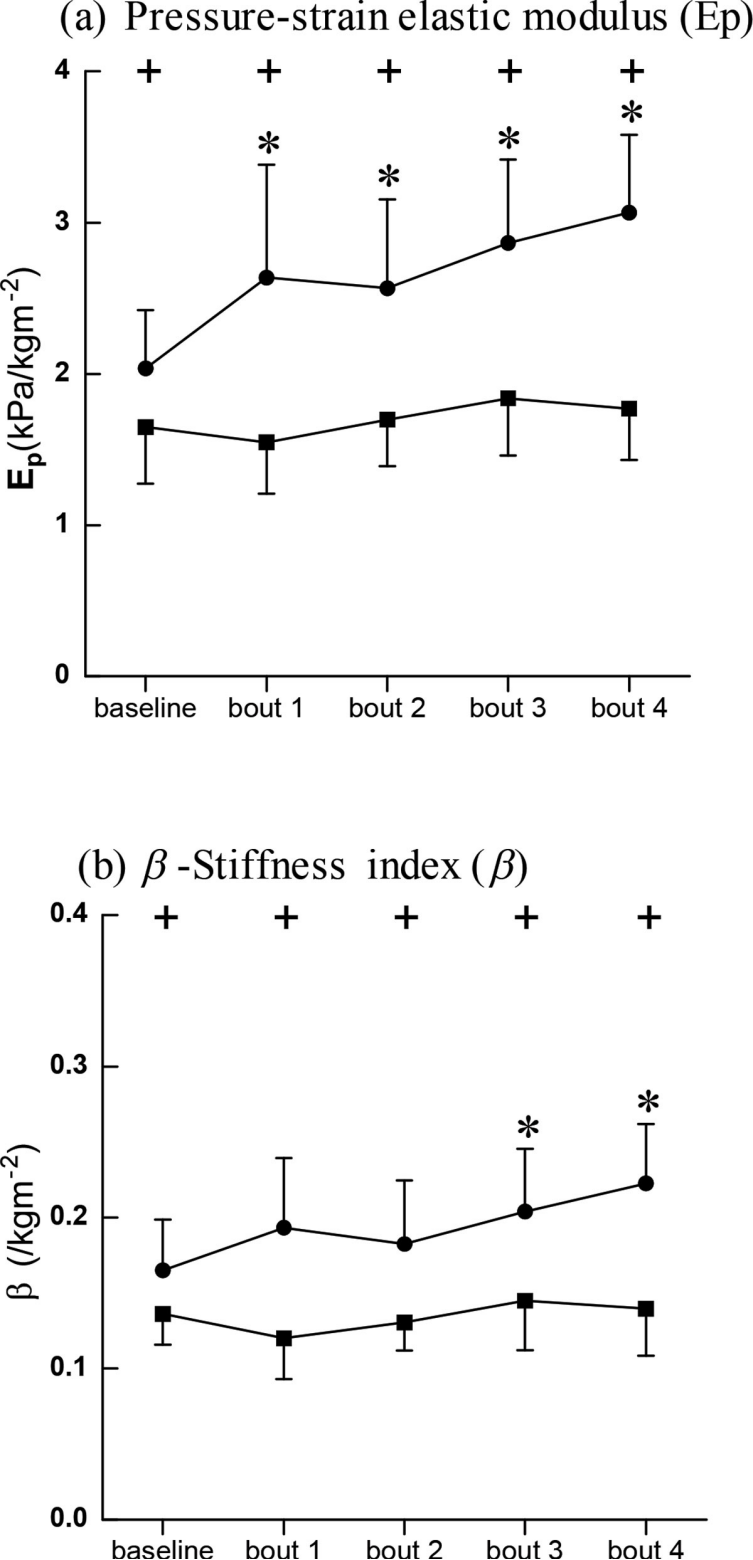
**Pressure-strain elastic modulus (E_P_) and *β*-Stiffness index (β)**. Data are presented for BA (■), as well as SC (●), + Significant difference between BA and SC: P < 0.05, * Significant difference from baseline within group: P < 0.05

As illustrated in Figure 2, at baseline, both the pressure-strain elastic modulus and arterial stiffness were significantly lower in BA than in SC.

**Figure 3:**
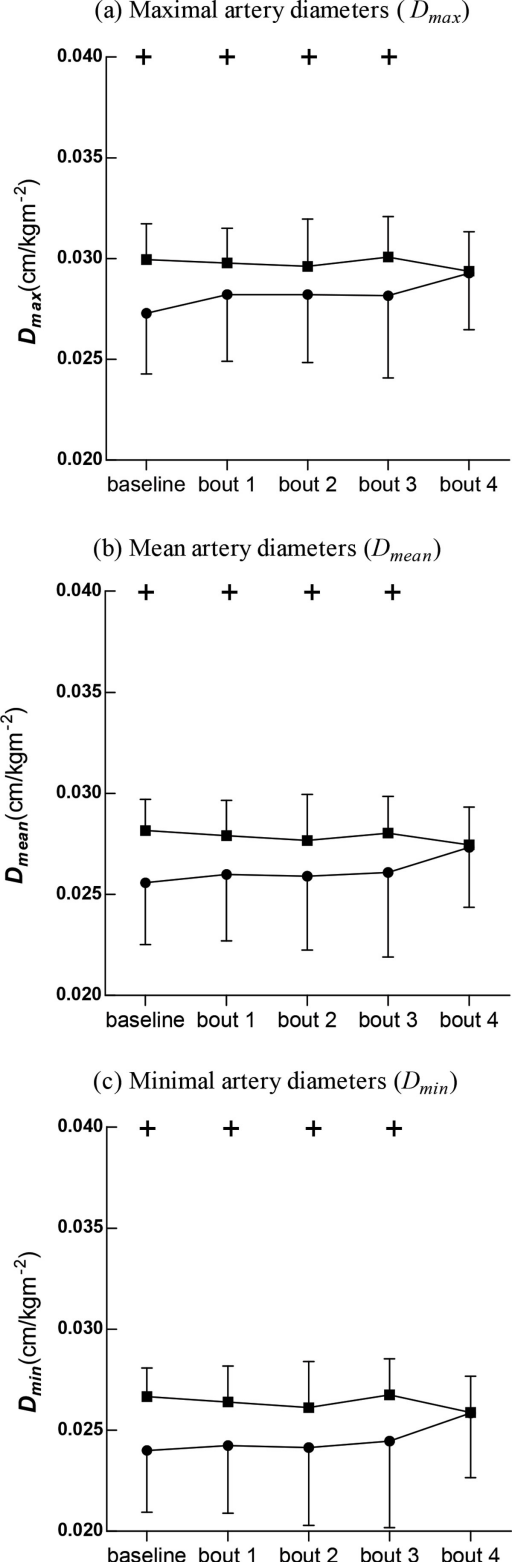
**Carotid artery diameters (D)**. Data are presented for BA (■), as well as SC (●), + Significant difference between BA and SC: P < 0.05, * Significant difference from baseline within group: P < 0.05

After the cycling intervention, the pressure-strain elastic modulus and arterial stiffness decreased slightly in BA at the first bout of cycling and then increased, but these changes were not statistically significant. In SC, however, the pressure-strain elastic modulus increased significantly while arterial stiffness increased gradually and showed significant differences at bouts 3 and 4. Figure 3 shows the changes in arterial diameters at baseline and immediately following the cycling intervention. It was evident that BA possessed significantly greater maximal, mean, and minimal arterial diameters compared with SC at baseline and after intervention with the exception of bout 4.

### Effect of cycling intervention on carotid blood supply to the brain

Table 1 shows the changes in heart rate, center-line velocity, and FR at baseline and immediately following the cycling intervention.

**Table 1:** Differences between BA and SC at baseline and right after exercise.

Variables	Subjects	Baseline	Cycling intervention			
			
			Bout 1	Bout 2	Bout 3	Bout 4
**HR**	BA	67 ± 6^+^	79 ± 10^+,*^	81 ± 9^+,*^	82 ± 9^+,*^	84 ± 4^+,*^
	SC	71 ± 9	99 ± 15^*^	106 ± 6^*^	110 ± 6^*^	113 ± 5^*^
***V _max_***	BA	51 ± 7^+^	59 ± 9^+,*^	68 ± 9^+,*^	63 ± 6^+,*^	63 ± 9^+,*^
	SC	71 ± 12	97 ± 22^*^	102 ± 17^*^	102 ± 16^*^	94 ± 25^*^
***V_mean_***	BA	20 ± 3^+^	23 ± 3^+,*^	26 ± 4^*^	25 ± 3^+,*^	24 ± 3^+,*^
	SC	27 ± 5	32 ± 7^*^	32 ± 8^*^	32 ± 6^*^	32 ± 7^*^
***V_min_***	BA	10 ± 5^+^	10 ± 4	7 ± 6	8 ± 5	7 ± 5^+,*^
	SC	14 ± 3	14 ± 8	10 ± 4^*^	9 ± 2^*^	10 ± 5
***Q_max_***	BA	12.82 ± 2.79	13.16 ± 3.23	15.43 ± 4.15^*^	15.86 ± 3.68^*^	16.20 ± .86^*^
	SC	11.82 ± 2.70	16.18 ± 5.45^*^	18.01 ± 6.87^*^	19.16 ± 9.82^*^	19.67 ± 7.41^*^
***Q_mean_***	BA	3.89 ± 0.62^+^	4.15 ± 0.52	4.45 ± 0.99	4.38 ± 0.93	4.01 ± 0.83
	SC	3.21 ± 0.57	3.79 ± 0.92^*^	3.95 ± 1.54	4.26 ± 1.97	4.40 ± 1.54
***Q_min_***	BA	0.90 ± 1.05	-0.39 ± 1.08	-1.05 ± 1.01^*^	-1.24 ± 1.07^*^	-1.57 ± 1.04^*^
	SC	0.94 ± 0.36	0.14 ± 1.15	-0.77 ± 1.18^*^	-1.11 ± 1.85^*^	-1.44 ± 1.23^*^

**HR **Heart rate (beats/min); ***V _max_***, ***V_mean_***, ***V_min _***maximal, mean, minimal center-line velocity (cm/s);

***Q_max_***, ***Q_mean_***, ***Q_min _***maximal, mean, minimal flow rate (ml/s).

^+ ^Significant difference between BA and SC: *P *< 0.05

^* ^Significant difference from baseline within group: *P *< 0.05

After the cycling intervention, heart rates significantly increased in both BA and SC, but increases among BA were significantly lower than those in SC both at baseline and immediately following the intervention. Center-line velocities were significantly higher in SC both at baseline and after intervention. After the cycling intervention, the maximal and mean center-line velocities increased significantly in both groups relative to baseline. Both BA and SC showed increased maximal and mean FRs ****and decreased minimal FRs after the cycling intervention compared with baseline. At baseline, the mean FR was significantly higher in BA than in SC. There were no significant differences between BA and SC after the intervention.

### Effect of cycling intervention on carotid hemodynamic forces

**Figure 4:**
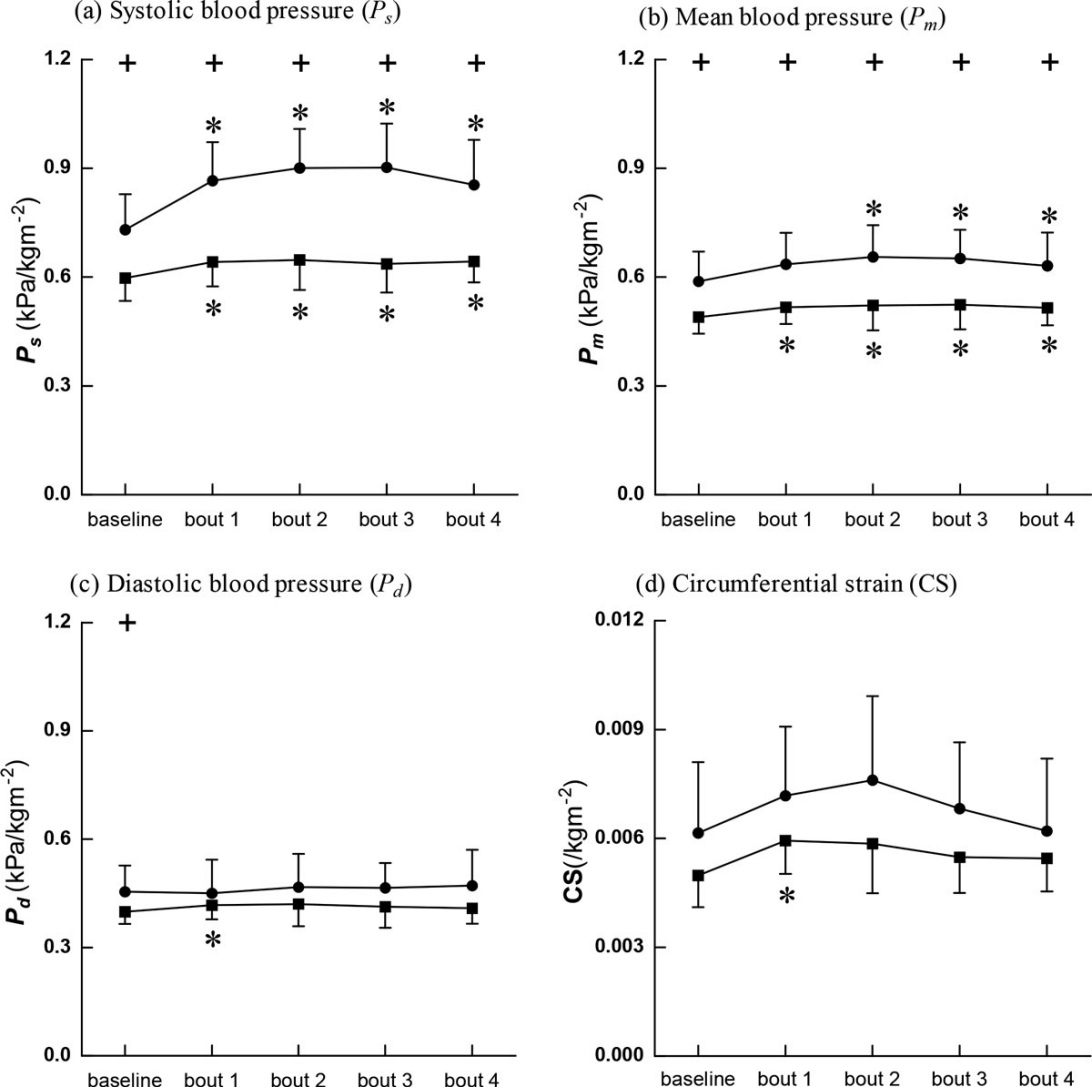
**Blood pressure and circumferential strain (CS)**. Data are presented for BA (■), as well as SC (●), + Significant difference between BA and SC: P < 0.05, * Significant difference from baseline within group: P < 0.05

Blood pressure data are shown in Figure 4(a-c). At baseline, systolic, diastolic, and mean blood pressures in BA were significantly lower than those in SC. After the cycling intervention, systolic and mean blood pressures in BA remained significantly lower than those in SC even though systolic, diastolic, and mean blood pressures in both groups tended to increase. CS (Figure 4d) was lower in BA than in SC both at baseline and after the intervention, but the differences were not significant.

**Figure 5:**
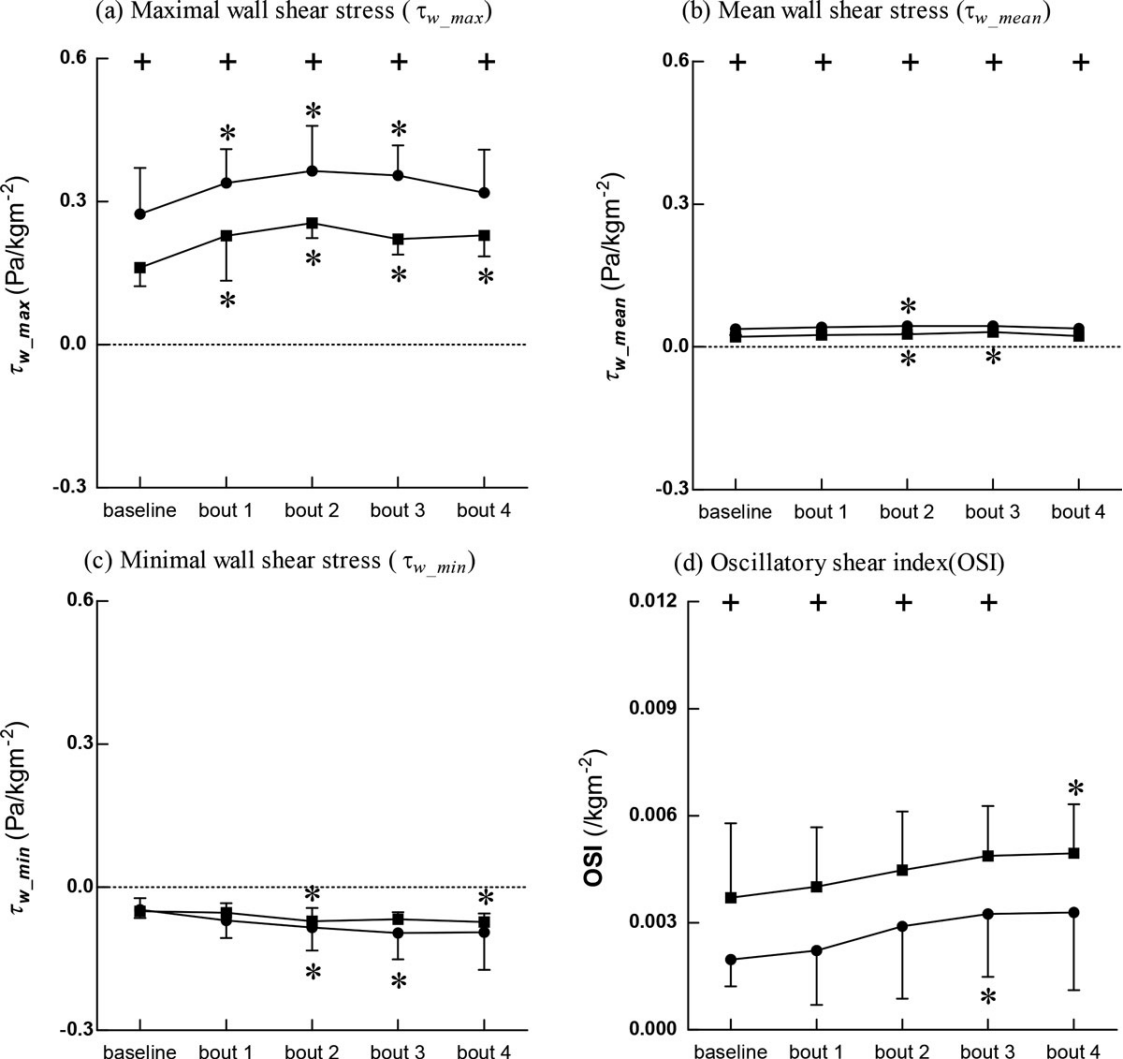
**Wall shear stress (WSS) and Oscillatory shear index (OSI)**. Data are presented for BA (■), as well as SC (●), + Significant difference between BA and SC: P < 0.05, * Significant difference from baseline within group: P < 0.05

Figure 5(a-b) shows that the maximal and mean WSS values in BA were significantly lower than those in SC both at baseline and after the intervention. The minimal WSS (Figure 5c) in SC was lower than that in BA after the cycling intervention, but the difference did not reach statistical significance. OSI (Figure 5d) was significantly higher in BA than in SC both at baseline and after the intervention.

## Discussion

Our results show that compared with SC, BA possess decreased stiffness and enhanced resting diameters in the carotid artery. In response to the accumulative effect of an acute cycling intervention, arterial stiffness in BA was hardly affected while that in SC continuously increased.

At baseline, decreased carotid arterial stiffness and an enlarged carotid arterial diameter were found in long-term trained BA compared with SC. Decreased arterial stiffness has been observed in endurance trained athletes (e.g., cyclists, distance runners) [10,25,26] as well as in young [27], middle-aged, and older-aged [28] regular aerobic exercisers while increased arterial stiffness was reported in young high-intensity resistance trained subjects [12] and in strength-exercising athletes [29]. Moreover, several studies [30,31] have shown that endurance-trained athletes have larger arteries than control subjects. Among sedentary men, an enhanced resting femoral artery diameter was observed after walking training [32].

At baseline, heart rates and center-line velocities were significantly lower in BA than in SC, but the mean FR was significantly higher in BA than in SC, demonstrating improved blood supply to the brain in BA. It was reported that resting stroke volume was greater in strength- and endurance-trained men [30], and that individuals with regular physiological activity also had higher cerebral blood flow than age-matched SC [33]. The results of this study confirmed that long-term basketball exercise led to an efficient resting blood supply. Hemodynamic variables including blood pressure and maximal and mean WSS were significantly lower in BA than in SC. However, the OSI was significantly higher in BA than in SC. It is well known that regular aerobic exercise reduces resting blood pressure and improves endothelium-dependent vasodilatation in hypertensive patients [34] as well as in healthy subjects [6]. Similarly, our results showed that resting blood pressure in long-term trained BA was reduced. In addition, it has been shown that changes in shear stress pattern induced the remodeling of arterial structure and function [27,35,36]. Our results indicated that maximal and mean WSS in BA are significantly lower than those in SC, and that the OSI in BA was significantly higher than that in SC. The roles of maximal and mean WSS and of the OSI with respect to changes in arterial structure and function in BA require further investigation.

After 4 bouts of cycling exercise, carotid arterial stiffness in BA exhibited no obvious changes whereas that in SC increased significantly. These results indicated that the more compliant vasculature of long-term trained BA has a greater capacity for resisting acute external intervention. In addition, hemodynamic variables in both BA and SC changed dynamically, but these changes exhibited different dynamic behaviors. While SC had higher heart rates and center-line velocities, BA had relatively lower heart rates and center-line velocities. Cycling intervention increased systolic and mean blood pressures in both BA and SC, but both were significantly lower in BA than in SC. A previous report showed that elevated blood pressures induced by acute resistance exercise may alter arterial structure and/or arterial load-bearing properties, resulting in increased arterial stiffness [5]. Our current results showed that long-term trained basketball players had decreased carotid blood pressure. After the cycling intervention, the maximal WSS in SC was higher than that in BA while the minimal WSS in SC was much lower than that in BA. However, the OSI remained significantly higher in BA than in SC.

A previous investigation [27] proposed that both low (or retrograde) shear stress and high shear stress may lead to endothelial damage and a subsequent pathological response. Yet, in this study, the maximal WSS in SC was larger than that in BA at baseline and after the cycling intervention, and the minimal WSS in SC was smaller than that in BA after the cycling intervention. As arterial stiffness in BA was lower than that in SC, the conflicting results require further studies to determine the relationship between the WSS pattern and arterial function after long-term basketball training and acute cycling intervention. It is also worth noting that the OSI was significantly higher in BA than in SC both at rest and after the intervention. It was well established by Ku et al. [23] that a low and oscillatory WSS with a higher OSI may enhance atherogenesis. However, this was doubted by some investigators [37] as the conclusions were mainly drawn from human post-mortem material, animals, and patients instead of healthy humans under rest conditions. In the present study, both at baseline and after the cycling intervention, BA had much higher OSI values. After exercise training, the heart rate would increase, which raises a challenging question that warrants further investigation, i.e., what is the role of an exercise-induced high OSI along with an increasing heart rate with respect to changes in arterial structure and function?

It has been estimated that 9% of all cardiovascular disease cases could be avoided if sedentary individuals became moderately active [6]. Dullness and lack of fun of aerobic [8] or endurance exercise [9-11] represents a major barrier to physical activity and has been associated with low physical activity levels. However, basketball training is a unique form of exercise that differs from aerobic and resistance modes of exercise. Basketball training involves endurance, speed, power and strength elements, as well as basketball skills. Basketball is also an exciting game that is attracting greater numbers of participants, especially from among younger populations. Our results demonstrated that basketball exercise decreased stiffness and enhanced resting diameters in the carotid artery. Therefore, basketball exercise can be recommended to young sedentary persons for their arterial health.

College BA rather than ordinary basketball players were recruited to participate in this study in order to enroll subjects who performed only basketball training and no other regular exercise. However, colleges BA are different with regard to height, mass, and other parameters. In this study, hemodynamic formulas were used to calculate dynamic parameters such as WSS and OSI. According to the equations (5-9) [21,23], arterial diameters are closely associated with subjects' stature including weight and height. Had we recruited ordinary basketball players, we could not guarantee their freedom from other physical activity. To minimize the size effect, BMI [24] was used to standardize the dynamic parameters and arterial diameter.

## Conclusion

In this study, long-term trained BA was found to possess decreased stiffness and enhanced resting diameters in the carotid artery compared with SC. In response to the accumulative effect of a cycling intervention, arterial stiffness in BA was hardly affected while that in SC increased continuously. At baseline and after the cycling intervention, local hemodynamics in BA and SC had different dynamic characteristics. It is worth noting that WSS patterns were quite different between BA and SC at baseline and after the cycling intervention. In particular, OSI values in BA were higher than those in SC, and further investigation is required to clarify their roles in the remodeling of arterial structure and function.

## Abbreviations

BA, Basketball athletes; SC, Sedentary controls; E_p_, Pressure-strain elastic modulus; *β*, *β*-Stiffness index; HR, Heart rate, FR, Flow rate; WSS, Wall shear stress; BP, Blood pressure; *P_max_*, *P_mean_*, *P_min_*, The values of maximal, mean and minimal blood pressure; *V_max_*, *V_mean_*, *V_min_*, The values of maximal, mean and minimal center-line velocity; *Q_max_*, *Q_mean_*, *Q_min_*, The values of maximal, mean and minimal flow rate; *D_max_*, *D_mean_*, *D_min_*, The values of maximal, mean and minimal carotid arterial diameter; *τ_w-max_*, *τ _w-mean_*, *τ _w-min_*, The values of maximal, mean and minimal wall shear stress; CS, Circumferential strain; OSI, Oscillatory shear index; ANCOVA, Analysis of covariance; rmANOVA, Repeated measures analysis of variance.

## Competing interests

The authors declare that they have no competing interests.

## Authors' contributions

HBL, WXY, and KRQ designed the study. HBL and JH conducted the experiments and collected the experimental data. HBL and KRQ wrote the manuscript. All authors read and approved the final manuscript.

## Author' details

Qin's group has been researching the regulation of arterial function via exercise intervention and associated hemodynamic mechanisms.

## Declarations

Publication of this article was paid with funding from National Natural Science Foundation of China, No. 31370948.

This article has been published as part of *BioMedical Engineering OnLine *Volume 14 Supplement 1, 2015: Cardiovascular Disease and Vulnerable Plaque Biomechanics. The full contents of the supplement are available online at http://www.biomedical-engineering-online.com/supplements/14/S1
